# Investigation of the Visible Photocatalytic–Fenton Reactive Composite Polishing Process for Single-Crystal SiC Wafers Based on Response Surface Methodology

**DOI:** 10.3390/mi16040380

**Published:** 2025-03-27

**Authors:** Zijuan Han, Bo Ran, Jisheng Pan, Rongji Zhuang

**Affiliations:** 1 School of Electromechanical Engineering, Guangdong University of Technology, Guangzhou 510006, China; 2 Han’s Laser Technology Industry Group Co., Ltd., Shenzhen 518000, China; 3 State Key Laboratory for High Performance Tools, Guangdong University of Technology, Guangzhou 510006, China

**Keywords:** SiC, core–shell structure, visible photocatalysis, response surface method, chemical mechanical polishing

## Abstract

The third-generation semiconductor single-crystal silicon carbide (SiC), as a typical difficult-to-machine material, improves the chemical reaction rate on the SiC surface during the polishing process, which is key to realizing efficient chemical mechanical polishing (CMP). In this paper, a new core-shell structure Fe_3_O_4_@MIL-100(Fe) magnetic catalyst was successfully synthesized, which can effectively improve the reaction rate during the SiC polishing procesSs. The catalyst was characterized by X-ray diffraction (XRD), scanning electron microscopy (SEM), and X-ray photoelectron spectroscopy (XPS), and was used as a heterogeneous photocatalyst for chemical mechanical polishing, and the polishing results of SiC were optimized using response surface methodology (RSM). The experimental results show that the surface roughness of SiC can reach the minimum value of 0.78 nm when the polishing pressure is 0.06 MPa, the polishing speed is 60 rpm, and the polishing flow rate is 12 mL/min. The results of the study provide theoretical support for the visible photocatalysis-assisted CMP of SiC.

## 1. Introduction

SiC has emerged as the most promising third-generation semiconductor material due to its exceptional physical and chemical properties, including a wide bandgap, high saturated electron drift velocity, high breakdown electric field strength, and high thermal conductivity. It is widely used in fields such as power electronics, optoelectronics, and high-temperature devices. In contrast to traditional semiconductors, such as silicon, SiC exhibits high thermal conductivity, exceptional mechanical strength, and outstanding chemical inertness [1,2]. Consequently, SiC devices offer superior performance, reliability, and efficiency, making them indispensable in various industries, including automotive, aerospace, and renewable energy [3]. The surface quality of the SiC material is critical to the performance of SiC-based devices, and chemical mechanical polishing (CMP) is a widely used planarization method for semiconductor wafers. CMP utilizes the combined effects of chemical reactions and mechanical removal to achieve surface polishing, effectively improving both polishing efficiency and quality [4].

It has been shown that the Fenton reaction based on advanced oxidation techniques can improve the CMP efficiency of SiC [5,6]. Among them, advanced oxidation processes (AOPs) based on the in situ generation of reactive oxidizing radicals are considered a promising technology for degrading pollutants. Among the AOPs, the heterogeneous photo-Fenton process has attracted much attention due to its strong generation of active radicals and ease of operation. Lu et al. [7] verified the synergistic effect of the heterogeneous photocatalyst through CMP experiments, in which the concentration of ·OH increased significantly in the composite reaction, and the material removal rate (MRR) reached 387.2 nm/h. Zhou et al. [8] evaluated the polishing performance of abrasive slurries containing SiO_2_@TiO_2_ composite nanoparticles under UV light. The MRR of the abrasive slurry containing SiO_2_@TiO_2_ composite nanoparticles was much higher than that of the slurry without SiO_2_@TiO_2_ particles. The MRR of SiC wafers reached approximately 120 nm/h. In photocatalytic-assisted chemical-mechanical polishing, UV photocatalysis mainly relies on the excitation of broad-band semiconductors such as TiO₂ with high-energy UV light (<400 nm) to generate strong oxidizing holes (h⁺) and free radicals (·OH), which assist in removing the surface of the material through oxidation reactions. Although UV photocatalysis is highly reactive and has a strong oxidizing ability, it can lead to non-selective surface damage such as over-etching, and it requires high-pressure mercury lamps or UV LEDs, which have high energy consumption and risk ozone generation. In contrast, visible-light photocatalysis utilizes low-energy visible light (400–700 nm), which can narrow the forbidden bandwidth by catalyst modification, such as nitrogen-doped TiO₂, g-C₃N₄, etc., to achieve the generation of elec-tron-hole pairs under mild conditions, with a mild reaction and a controllable oxidizing ability, which is more suitable for polishing applications. Therefore, the use of visible light photocatalysis-assisted chemical-mechanical polishing not only solves the problem of the expensive and short lifetime of the UV light source equipment, but also avoids the UV-induced high toxicity by-products such as chlorine-containing radicals and also avoids the UV-induced peroxidation reaction, reduces the micro-damage of the surface of the substrate, and improves the uniformity of the polishing, which is of great significance for photocatalytic-assisted polishing. Yang et al. [9] successfully combined nanodiamond (ND) and copper–iron layered double hydroxide (LDH) to prepare composites for visible photocatalysis. Experimental results showed that under visible light irradiation, the solution containing ND/LDH exhibited the highest ·OH concentration, which effectively enhanced the oxidizing properties of the polishing solution. This composite was successfully used in the CMP of single-crystal diamonds, achieving high-quality polishing under visible light.

Among various photocatalytic materials, metal-organic frameworks (MOFs) are commonly used in wastewater treatment due to their high specific surface area, tunable pore sizes, and diverse chemical compositions. These materials feature a highly porous network structure with well-defined pores and a large surface area, making them ideal for various environmental remediation and catalytic reactions [10]. The modular nature of MOFs allows for precise control over their structure and properties, enabling the design of materials with tailored characteristics for specific applications. Sijia et al. [11] rapidly, in just 30 min, prepared Fe_3_O_4_@MIL-100(Fe) using a microwave method and used it as an adsorbent and photocatalyst for the removal of diclofenac sodium (DCF) from water. The results showed that the composite structure of Fe_3_O_4_@MIL-100(Fe) possessed higher stability, reactivity, and tunable surface properties, removing 87.8% of DCF. The transition of CMP of SiC from UV to visible photocatalysis provides an opportunity to overcome the limitations of conventional UV photocatalysis. The focus of this study is to synthesize an inexpensive catalyst that can be combined with a visible LED light source and to investigate the synergistic effect of visible-light photocatalysis and mechanical polishing to dynamically regulate the polishing rate for a more efficient, cost-effective, and environmentally friendly CMP process on silicon carbide substrates.

## 2. Experimental Materials and Methods

### 2.1. Synthesis of Fe_3_O_4_@MIL-100(Fe)

**Figure 1:**
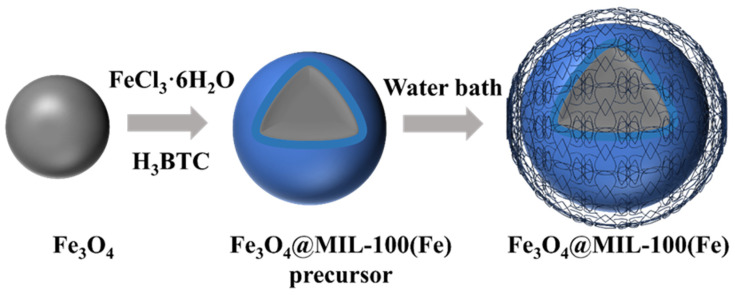
Simple diagram of the synthesis steps of Fe_3_O_4_@MIL-100(Fe).

The synthesis steps of Fe_3_O_4_@MIL-100(Fe) are shown in Figure 1. Composite particles were prepared according to the method of Aslam [12,13,14] with some modifications. 1.0 g Fe_3_O_4_ (particle size: 200 nm, Macklin, Shanghai, China) was dispersed in an ethanol solution of FeCl_3_–6H_2_O (Ke Rong Biological Co., Guangzhou, China) at a concentration of 120 mmol/L and stirred at 60 rpm for 15 min, then separated magnetically with neodymium magnets and cleaned with ethanol, and the collected particles were added to an ethanol solution of H_3_BTC (J&K Scientific, Beijing, China) at a concentration of 132 mmol/L and stirred at 60 rpm for 15 min. After obtaining the composite particulate precursor, the solution was transferred to a reactor lined with PTFE (LICHEN, model LC-KH-200, Guangzhou, China) and heated at 70 °C for 12 h. At the end of the reaction, the reactor was cooled to room temperature, and the particles were recovered by applying a magnetic field. To remove impurities from the surface of the composite particles, the synthesized composite particles were washed three times with ethanol for further purification, then dried under vacuum at 70 °C for 12 h and milled for recovery. The final magnetic metal-organic skeleton composites were obtained.

### 2.2. Characterization of Fe_3_O_4_@MIL-100(Fe)

The surface morphology of the material was characterized using a scanning electron microscope (SEM, Apreo 2S HiVac, Thermo, Waltham, MA, USA), and the powder particles were uniformly dispersed on a conductive carbon tape, and then gold sprayed. Particle size analysis was performed using ImageJ software (v1.53). X-ray diffraction patterns were recorded using an X-ray diffractometer (D8 ADVANCE, Bruker, Berlin, Germany). The surface chemical state information for the materials was characterized using an X-ray photoelectron spectroscopy system (ESCALAB 250Xi, Thermo, Waltham, MA, USA).

### 2.3. SiC Chemical Mechanical Polishing Test

**Figure 2:**
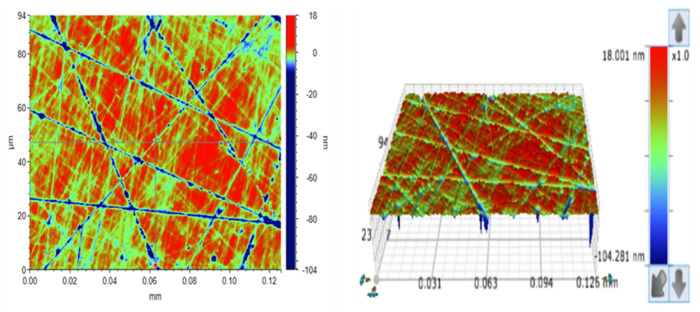
Surface morphology of the SiC crystals before polishing.

**Figure 3:**
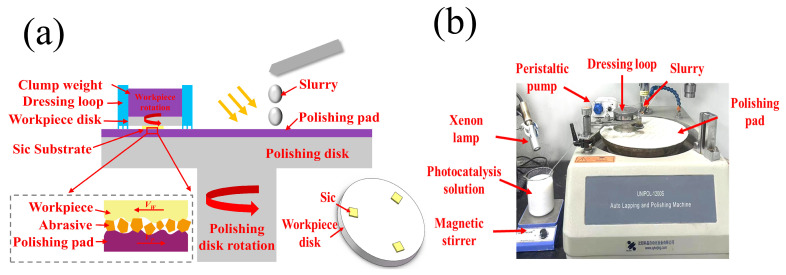
Scheme of the CMP experiment (**a**) and the equipment (**b**).

A SiC wafer with dimensions of 10 mm × 10 mm was used for the chemical mechanical polishing (CMP) experiments. The surface morphology of SiC after grinding is shown in Figure 2, where it can be observed that numerous deep scratches were present on the SiC surface, with the maximum scratch depth of 100 nm and the surface roughness (Ra) of approximately 2–3 nm. The principle of CMP and the associated equipment are illustrated in Figure 3. Before polishing, the SiC wafer was fixed onto the workpiece disk using paraffin wax. The workpiece disk, with the SiC wafer in place, was then positioned in a dressing loop, and weights were applied to adjust the polishing pressure, ensuring full contact between the workpiece disk and the polishing pad. Under the rotation of the polishing disk, relative motion was generated between the SiC wafer and the abrasive material on the polishing pad, effectively removing the oxide layer from the surface. After polishing, the SiC wafer was immersed in deionized water, ultrasonically cleaned for 5 min, and weighed using a precision electronic balance. The material removal rate (MRR) of SiC wafers in each CMP test was calculated using Equation (1).

(1) $$MRR=\frac{m_{0}-m_{1}}{\rho \times S\times T}$$

where *m*_0_ is the mass of SiC before polishing and *m*_1_ is the mass after polishing (in mg) weighed with a precision balance with a precision of 0.1 mg; SiC wafer density, ρ = 3.2 g/cm^3^; S is the area of SiC, mm^2^; *T* is the processing time, h; and the units of MRR are nm/h.

The surface roughness of SiC before and after polishing was detected using a white light interferometer (BRUKER Contour GT-X, Bruker, Berlin, Germany), and to improve the reliability of the data, five different areas of the SiC surface were detected separately, and three measurements were taken in.

## 3. Results and Discussion

### 3.1. Characterization Results of Fe_3_O_4_@MIL-100(Fe)

**Figure 4:**
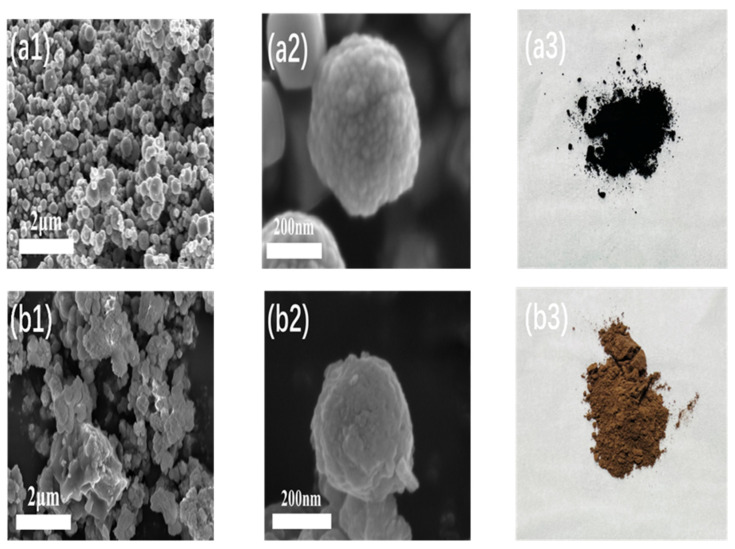
SEM images of Fe_3_O_4_ (**a1**,**a2**) and Fe_3_O_4_@MIL-100(Fe) (**b1**,**b2**), powder samples of Fe_3_O_4_ (**a3**) and Fe_3_O_4_@MIL-100(Fe) (**b3**).

**Figure 5:**
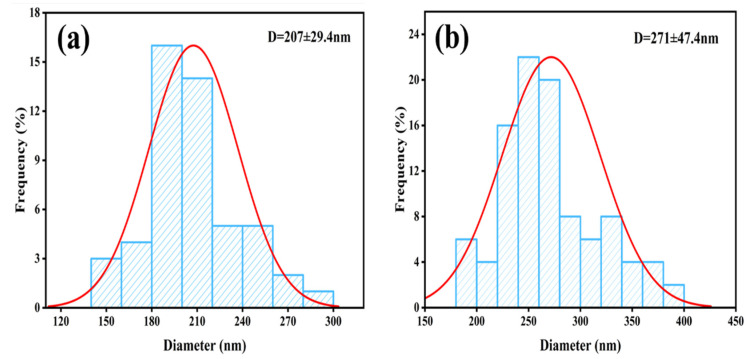
Particle size distribution of Fe_3_O_4_ (**a**) and Fe_3_O_4_@MIL-100(Fe) (**b**).

Figure 4(a1–a3) shows the SEM images and powder samples of Fe_3_O_4_, respectively. It can be observed that the Fe_3_O_4_ particles were uniformly distributed in size, presented a spherical shape, their surface was relatively smooth, and the color of the powder sample was black. Figure 4(b1–b3) shows the SEM images and powder samples of Fe_3_O_4_@MIL-100(Fe), and Figure 5 shows the particle size distribution of Fe_3_O_4_ and Fe_3_O_4_@MIL-100(Fe). The surface of the composite particles was rougher compared with that of Fe_3_O_4_, with particle sizes between 220–390 nm, and the surface of the catalyst appears to be wrinkled with more obvious layer-like substances because MIL-100(Fe) grew on Fe_3_O_4_ microspheres [14], wrapping them layer by layer in a MOF structure. Thus, the surface of the composite material catalyst had more layered material, and the color of the powder sample appeared to be brown. This alteration also proved the success of the preparation of the core-shell structure initially.

**Figure 6:**
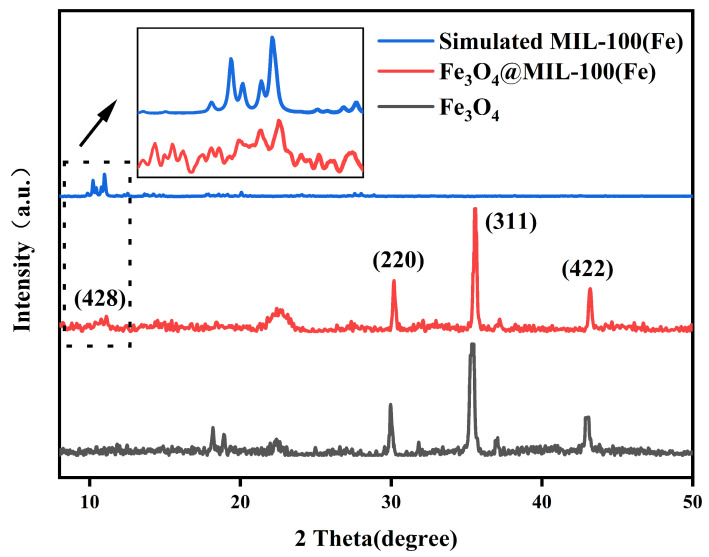
XRD images of Fe_3_O_4_, MIL-100(Fe), and Fe_3_O_4_@MIL-100(Fe).

To further investigate the structure of the synthesized composite particles, X-ray diffraction (XRD) was employed for characterization. The XRD results, presented in Figure 6, show the spectra of Fe_3_O_4_, MIL-100 (Fe), and the composite particles. As observed, the diffraction peaks of the synthesized composite particles align with those of Fe_3_O_4_ and MIL-100 (Fe). The XRD pattern in Figure 6 reveals several diffraction peaks for the composite particles at 2θ = 11.0° (428), 30.2° (220), 35.4° (311), 43.3° (422). After the introduction of the shell material, the characteristic diffraction peak corresponding to MIL-100 (Fe) appeared near 11°, and although the intensity was weaker, the diffraction peaks observed in this study were consistent with those previously reported [15,16,17]. The XRD spectra further confirmed the successful synthesis of the magnetic metal-organic framework composites.

**Figure 7:**
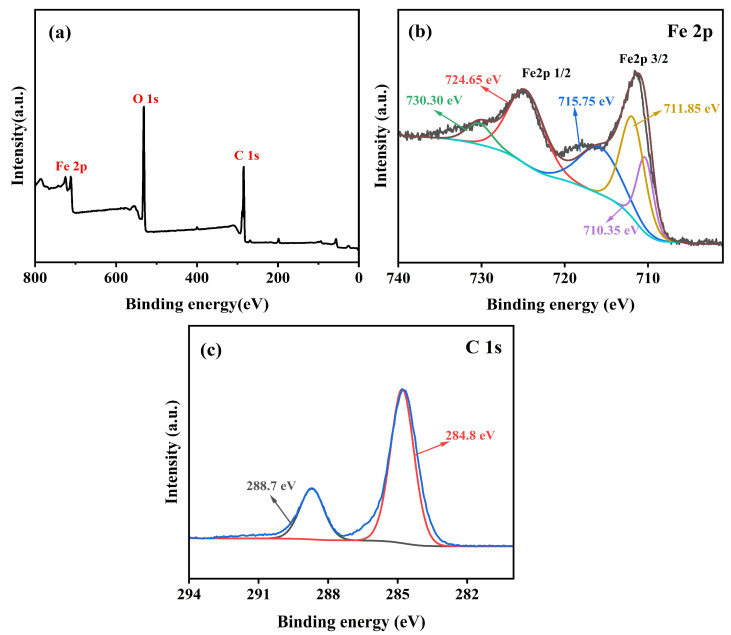
XPS images of Fe_3_O_4_@MIL-100(Fe): (**a**) full spectrum, (**b**) Fe 2p, and (**c**) C 1s.

To further determine the structure of the synthesized Fe_3_O_4_@MIL-100(Fe) material, the magnetic metal-organic framework composite was characterized using X-ray photoelectron spectroscopy (XPS). The results, presented in Figure 7, include complete spectroscopic measurements for Fe 2p, and C 1s, confirming the presence of the main elements in the MIL-100(Fe) layer, primarily Fe, and C. The Fe 2p spectrum shows two binding energy peaks at 711.85 eV (Fe 2p3/2) and 724.65 eV (Fe 2p1/2), which are separated by 12.8 eV, in agreement with previous literature on α-Fe2O3 [18], in addition to three satellite peaks at 710.35 eV, 715.7 5eV, and 730.30 eV attributed to Fe^3+^ in MIL-100 (Fe). Figure 7. c shows the XPS spectra for C. In the C 1s spectrum, the fitted peaks at c=o (288.7 eV), and c-c (284.8 eV) correspond to the benzene ring and carboxylic functional group of MIL-100(Fe) [19]. These states of Fe and C are consistent with the literature [20,21,22], further confirming the successful synthesis of the magnetic metal-organic framework composites.

### 3.2. Effect of Different Catalytic Conditions on Chemical Mechanical Polishing

**Figure 8:**
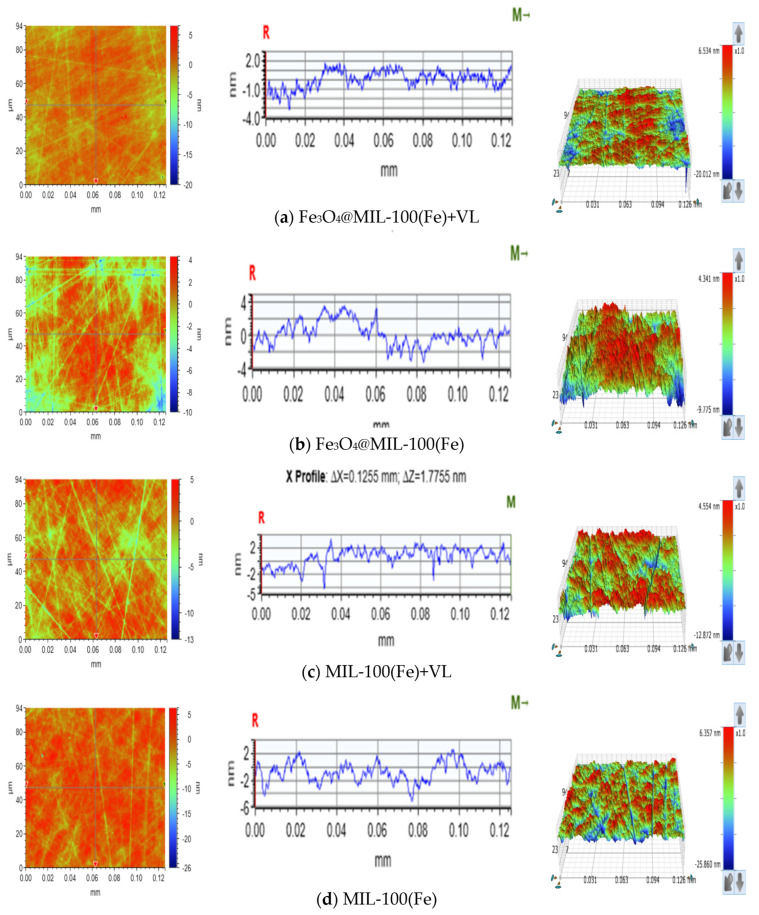

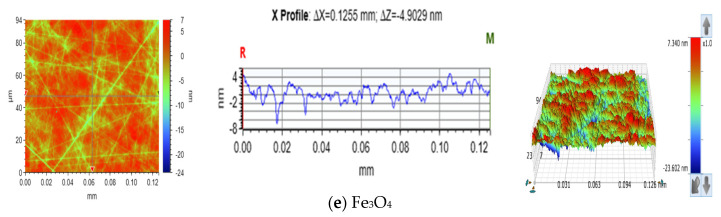
Surface morphology of SiC under different catalytic conditions.

**Figure 9:**
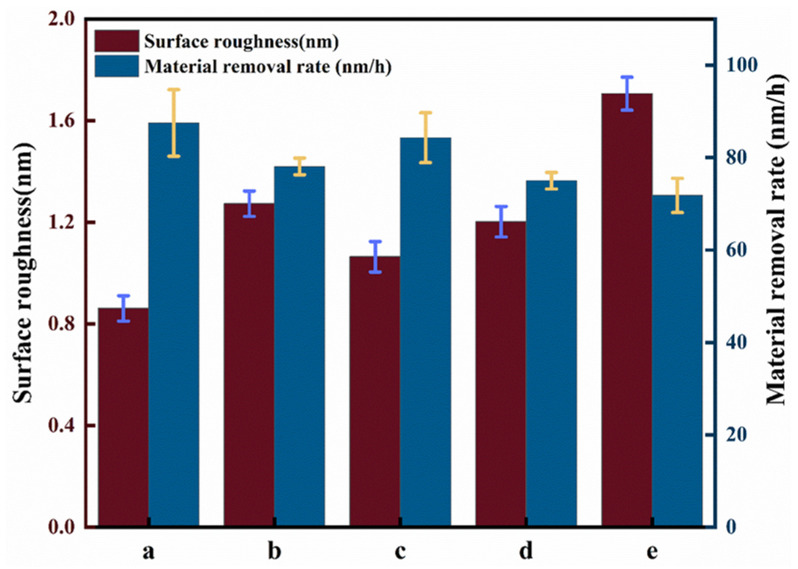
Effect of different catalysts on the experimental results of SiC.

The CMP experiments of SiC were conducted with the following parameters: an initial H_2_O_2_ concentration of 5 wt%, catalyst concentration of 14 g/L, light intensity of 500 W, pH 3, 20 wt% silica sol (100 nm), polishing speed of 50 rpm, polishing pressure of 0.06 MPa, and polishing time of 1 h. The results of the chemical mechanical polishing (CMP) experiments are presented in Figure 8 and Figure 9, which compare the visible photocatalytic–Fenton composite polishing of SiC wafers under different catalytic conditions. As shown in Figure 8 and Figure 9, compared to the original surface, the five groups of polished SiC in Figure 8 showed material removal, and the concave and convex peaks on the surface were reduced. Among these, the Ra of the visible photocatalytic–Fenton composite polishing group was 0.861 nm, with a smoothed surface where most of the scratches and rough peaks were removed. The deep pits present on the initial surface were also largely eliminated. In contrast, the Fenton group exhibited the worst surface morphology, with an Ra of 1.730 nm and deep scratches on the SiC surface, the deepest of which was 8 nm. Since the Mohs hardness of SiC is 9.5 and the hardness of silica sol is 7, the lower hardness of silica sol compared to SiC results in limited mechanical removal efficiency. In the case of the Fenton reaction, the weak chemical action prevented the production of sufficient ·OH to form a softened layer of lower hardness, and material removal was primarily dependent on abrasive action, leading to low efficiency. Consequently, the Ra of the Fenton reaction group did not decrease significantly. In contrast, visible photocatalytic Fenton composite polishing could continuously generate ·OH, which reacted with the SiC wafer to form a softer SiO_2_ oxide layer. This lowered the surface hardness of the SiC wafer, making it easier for the abrasives to remove the oxide layer, thereby eliminating the original scratches and defects. This resulted in an increased material removal rate and reduced surface roughness, leading to a smooth SiC surface [23,24]. The synergistic effect of visible photocatalytic and Fenton reaction composite polishing enhanced the polishing efficiency of SiC, yielding a better surface quality.

### 3.3. Analysis of Experimental Results of the Response Surface Method

**Figure 10:**
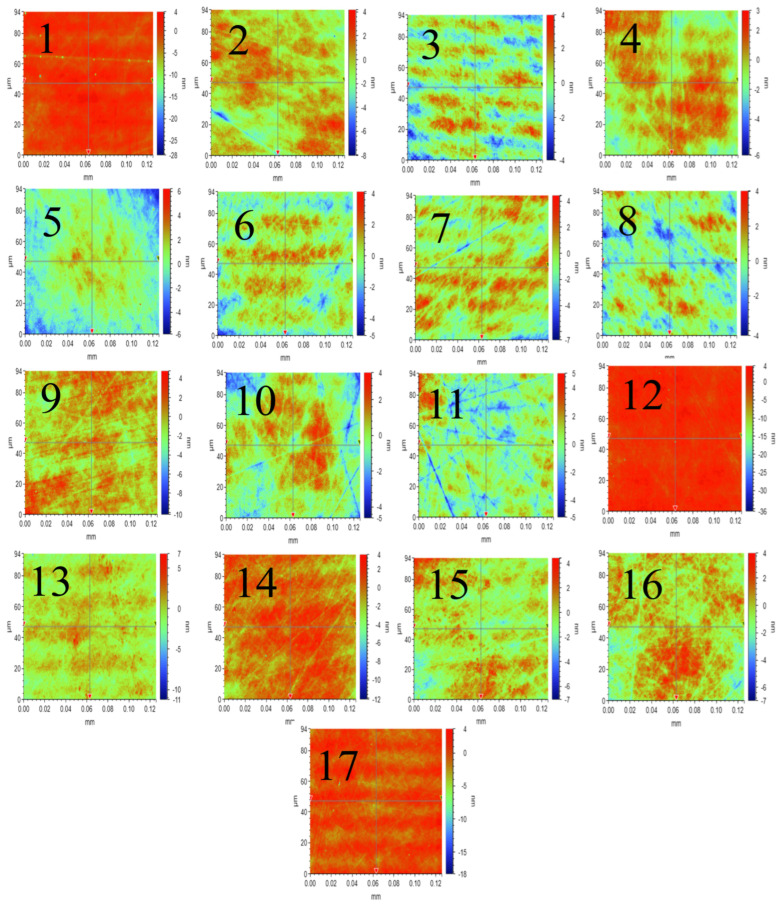
Experimental results of SiC surface topography based on response surface.

The process parameter intervals applicable to the CMP of SiC wafers were initially determined based on experimental results. Polishing pressure (A), polishing speed (B), and polishing liquid flow rate (C) were selected as the independent variables, with surface roughness (X) as the response variable. A response surface experimental design was employed, and the results were analyzed. During the design of the response surface experiment, the interval ranges of the independent variables were input into the Design-Expert software (v. DX13). The factors and levels for the response surface are shown in Table 1: the polishing pressure ranged from 0.03 to 0.09 MPa, the polishing speed—from 50 to 70 rpm, and the polishing flow rate—from 10 to 14 mL/min. The experimental design and results are presented in Table 2, while the results of the SiC surface topography experiments based on the response surface are shown in Figure 10.

After experimentally deriving the index value of the surface roughness of SiC under the above series of process parameters, the obtained results were imported into Design-expert (v. DX13), and the actual mathematical prediction model corresponding to the surface roughness was finally derived, as shown in Equation (2).

X = 8.60 + 1.56 × A + 0.9362 × B + 0.2350 × C − 0.2325 × AB − 0.0800 × AC − 0.0800BC − 0.1972 × A^2^ − 0.3572 × B^2^ − 0.5098 × C^2^ (2)

In Equation (2), A represents the polishing pressure, MPa; B represents the polishing disk rotation speed, r/min; C represents the polishing liquid flow rate, ml/min; and X represents the surface roughness, nm.

Table 3 presents the ANOVA results for the mathematical prediction model of surface roughness. The ANOVA analysis revealed that the *p*-value of the surface roughness model was 0.0018, indicating statistical significance, while the *p*-value for the out-of-fit term was 0.1306, suggesting that it was not significant. Therefore, the mathematical prediction model constructed based on the experimental results was all significant. The F-values for the polishing pressure, polishing speed, and polishing fluid flow rate were 49.61, 8.92, and 6.58, respectively. This indicates that the polishing pressure had the greatest influence on the surface roughness of SiC, followed by the polishing speed, with the polishing fluid flow rate having the least impact.

**Figure 11:**
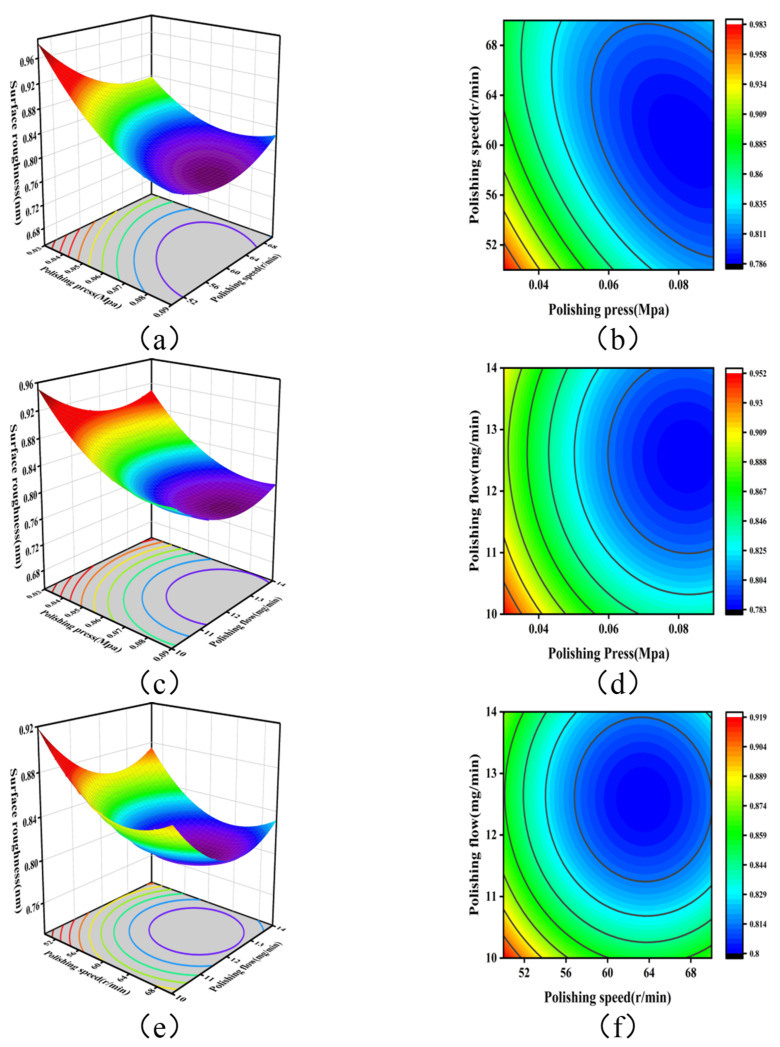
Response surfaces of the effects of process parameters on surface roughness: (**a**) AB interaction, 3D response surface; (**b**) AB interaction, contour plot; (**c**) AC interaction, 3D response surface; (**d**) AC interaction, contour plot; (**e**) BC interaction, 3D response surface; and (**f**) BC interaction, contour plot.

The response surface plots for the effects of the polishing pressure, polishing speed, and polishing fluid flow rate on the surface roughness of SiC are shown in Figure 11. Figure 11a,b illustrates the interaction between the polishing pressure and the polishing speed. As shown in Figure 11a, when the polishing pressure was within the range of 0.06–0.09 MPa, an increase in the polishing disk rotation speed initially decreased the surface roughness, which then increased again. In the significance analysis, the *p*-value for the interaction between the polishing pressure and the polishing speed was 0.0324, which is less than 0.05, indicating that this interaction was significant. Selecting appropriate values for both factors led to a more significant improvement in surface roughness. Figure 11c,d shows the interaction between the polishing pressure and the polishing fluid flow rate. In Figure 11c, the overall trend of the interaction response surface gradually declined. The *p*-value for the interaction between the polishing pressure and the polishing fluid flow rate was 0.8943, which is greater than 0.05, indicating that this interaction was not significant. Figure 11e,f displays the interaction between the polishing speed and the polishing fluid flow rate. From Figure 11e, it is evident that the response surface for the interaction between the polishing disk rotation speed and the polishing fluid flow rate showed a concave rise in the center. The *p*-value for this interaction was 0.8595, also greater than 0.05, indicating that the interaction was not significant. Figure 11f shows that there was an optimal surface roughness value in the interaction region of the polishing speed and flow rate. Figure 11 demonstrates that when either the polishing speed or the polishing fluid flow rate was held constant, the polishing pressure had a more significant effect on surface roughness. This finding is consistent with the ANOVA results of the regression model for surface roughness, which indicate that the polishing pressure had the greatest impact, followed by the polishing speed, with the polishing fluid flow rate having the least effect.

**Figure 12:**
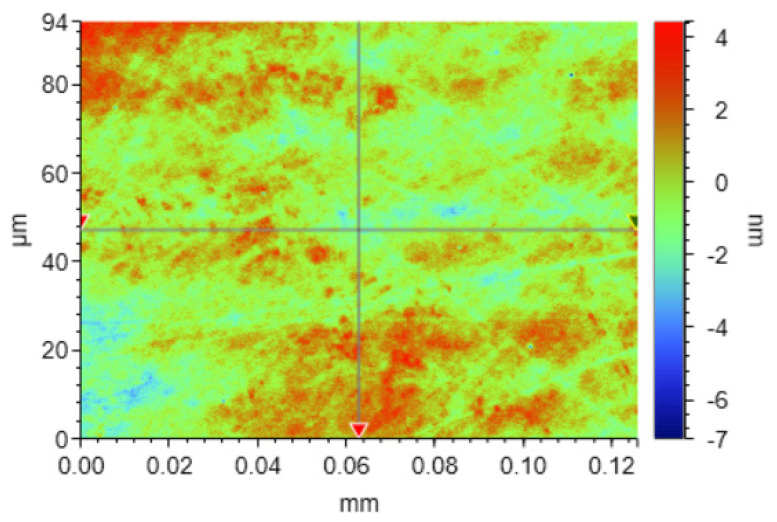
SiC surface morphology at the optimal parameters.

Based on the results of the response surface experiments, a mathematical prediction model for surface roughness was established. This model was then used to predict the optimal parameter ranges and, combined with the actual parameter settings, determine the optimal combination of process parameters. When the values of each process parameter were 0.059 MPa, 64.443 rpm, and 12.507 mL/min, the predicted surface roughness reached a minimum of 0.803 nm. The process parameters set in actual polishing were 0.06 MPa, 60 rpm and 12 mL/min. The results of the chemical mechanical polishing experiments using the actual process parameters were compared with the model’s predictions, as shown in Table 4. The experimental surface roughness value of 0.78 nm was very close to the predicted value of 0.803 nm, with an error of less than 5%, indicating the accuracy of the model’s predictions. Therefore, the optimal process parameters were determined to be a polishing pressure of 0.06 MPa, a polishing disk speed of 60 rpm, and a polishing solution flow rate of 12 mL/min. The surface morphology of the polished SiC wafer under these optimal conditions is shown in Figure 12. As seen in Figure 12, the surface roughness of the SiC wafer polished under these conditions was 0.78 nm, representing a significant improvement compared to the surface quality of the original SiC wafer.

### 3.4. Material Removal Mechanisms

**Figure 13:**
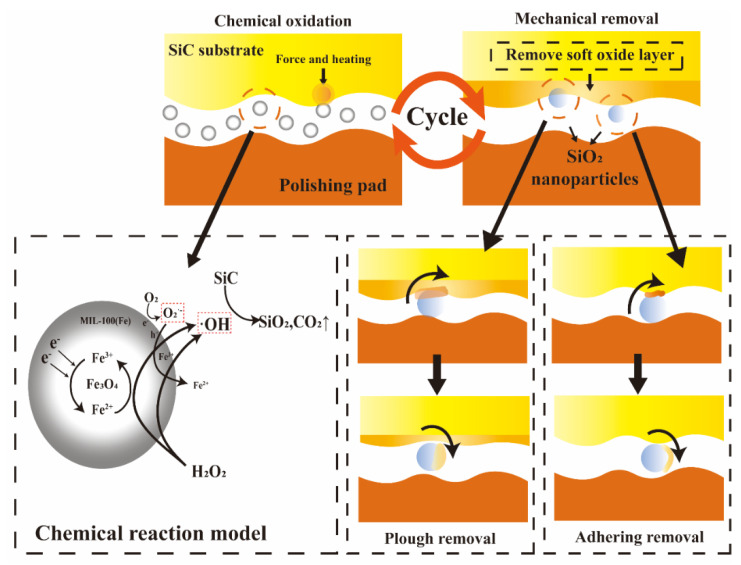
Simple material removal mechanisms.

The experimental results demonstrate that the oxidizing ability of the polishing solution was enhanced under the synergistic effect of visible light catalysis and the Fenton reaction. The material removal model of SiC under visible light catalysis is illustrated in Figure 13. There are two main sources of ·OH concentration in the chemical oxidation process: visible light catalysis and the Fenton reaction, with the chemical reactions occurring as shown in Equations (3)–(9) [7,14]. In Equation (3), the oxides formed on the SiC surface primarily depend on ·OH, and as the ·OH concentration increases, the generation of SiO_2_ also increases. Under visible light catalysis, the key chemical reactions occurring in the polishing solution are depicted in Equations (4)–(9). The Fe–O clusters in MIL-100(Fe) are activated by visible light [25], generating photogenerated electrons (e^−^) and photogenerated holes (h^+^) on the surface. Subsequently, due to the lower conduction band (CB) level of Fe_3_O_4_ compared to MIL-100(Fe), the e^−^ can transfer to the Fe_3_O_4_ surface, preventing the direct recombination of the photogenerated electron-hole pairs [11,26]. The e^−^ and h^+^ are then transported to H_2_O_2_ and Fe_3_O_4_, respectively, where the photogenerated electrons react with H_2_O_2_ and O_2_ to form ·OH and O_2_^−^, while the photogenerated holes react with H_2_O to produce ·OH. This ·OH can directly oxidize SiC, forming a softer oxide layer of SiO_2_, while H_2_O_2_ also generates ·OH in the presence of light. The introduction of visible light accelerates the catalytic process and increases the ·OH content in the polishing solution, thereby enhancing the formation of the SiC surface oxide layer. In the Fenton reaction, Fe_3_O_4_ acts as a nucleus to ionize Fe^2+^, which then reacts with H_2_O_2_ to produce ·OH and Fe^3+^. Fe^3+^ in the polishing solution is gradually converted back to Fe^2+^, thus enabling the continuous production of ·OH. Consequently, the visible photocatalytic and Fenton reactions work synergistically to enhance the oxidation process of the polishing solution, further increasing the generation of the SiC oxide layer. Additionally, the abrasives, through extrusion and relative motion with SiC, generate a flash point and release a significant amount of heat, with the temperature reaching over 1000 °C [27,28]. The higher flash point temperature reduces the activation energy required for the oxidation of the SiC surface, improving the formation of SiC surface oxides and achieving better polishing quality.

(3) $$SiC+4\cdot OH+O_{2}\to SiO_{2}+2H_{2}O+CO_{2}↑$$

(4) $$MIL-100\left(Fe\right)+h\nu \to h^{+}\left(MIL-100\left(Fe\right)\right)+e^{-}\left(MIL-100\left(Fe\right)\right)$$

(5) $$H_{2}O_{2}+e^{-}\left(\;Fe_{3}O_{4}@\;MIL-100\left(Fe\right)\right)\;\to 2\cdot OH$$

(6) $$O_{2}+e^{-}\to \cdot {O_{2}}^{-}$$

(7) $$h^{+}+H_{2}O\to \cdot OH+H^{+}$$

(8) $$Fe^{3+}+H_{2}O_{2}\to Fe^{2+}+\cdot OOH+H^{+}$$

(9) $$Fe^{2+}+H_{2}O_{2}\to Fe^{3+}+\cdot OH+OH^{-}$$

Finally, during the mechanical removal process, the abrasive primarily removes the oxide layer generated on the SiC surface through two mechanisms. Under the influence of pressure, the abrasive can embed itself into the oxide layer, leading to plowing removal, which results in the removal of a significant portion of the oxide layer. Additionally, through adhesive forces, the SiO_2_ abrasive removes the softened oxide layer by adhesion [29]. The cyclic interaction of chemical oxidation and mechanical removal allows the oxide layer on the SiC surface to be continuously removed by the abrasive, thereby improving the surface quality.

## 4. Conclusions

1. Fe_3_O_4_@MIL-100(Fe) was synthesized using a hydrothermal method, and the catalyst was fully characterized using an X-ray diffractogram, scanning electron microscopy, X-ray photoelectron spectroscopy, and the results showed the successful synthesis of novel core-shell structure Fe_3_O_4_@MIL-100(Fe) magnetic nanoparticles.
2. CMP experiments were carried out under different catalytic conditions, and the results showed that under the synergistic conditions of visible light catalyzed-Fenton reaction, the surface roughness of SiC reached an optimal value of 0.861 nm, which improved the surface quality by 50% compared to the Fenton group.
3. A mathematical prediction model for surface roughness was established. When the actual process parameters were as follows: polishing pressure of 0.06 MPa, polishing speed of 60 rpm, and polishing flow rate of 12 mL/min, the surface roughness achieved was as low as 0.78 nm.

## Figures and Tables

**Table 1:** Response surface factors and level values.

Level	Polishing Pressure (MPa)	Polishing Speed (r/min)	Polishing Flow Rate (mL/min)
−1	0.03	50	10
0	0.06	60	12
1	0.09	70	14

**Table 2:** Experimental design scheme and experimental results.

Num	A (MPa)	B (r/min)	C (mL/min)	X (nm)
1	0.03	50	12	0.983
2	0.09	50	12	0.842
3	0.03	70	12	0.856
4	0.09	70	12	0.831
5	0.03	60	10	0.968
6	0.09	60	10	0.831
7	0.03	60	14	0.921
8	0.09	60	14	0.790
9	0.06	50	10	0.906
10	0.06	70	10	0.879
11	0.06	50	14	0.867
12	0.06	70	14	0.848
13	0.06	60	12	0.809
14	0.06	60	12	0.811
15	0.06	60	12	0.783
16	0.06	60	12	0.816
17	0.06	60	12	0.823

**Table 3:** ANOVA results of the regression model for surface roughness.

Source	Sum of Squares	df	Mean Square	F-Value	*p*-Value	
Model	0.0508	9	0.0056	11.90	0.0018	significant
A	0.0235	1	0.0235	49.61	0.0002	
B	0.0042	1	0.0042	8.92	0.0203	
C	0.0031	1	0.0031	6.58	0.0373	
AB	0.0034	1	0.0034	7.09	0.0324	
AC	9.000 × 10^−6^	1	9.000 × 10^−6^	0.0190	0.8943	
BC	0.0000	1	0.0000	0.0337	0.8595	
A2	0.0055	1	0.0055	11.53	0.0115	
B2	0.0047	1	0.0047	9.99	0.0159	
C2	0.0046	1	0.0046	9.69	0.0170	
Residual	0.0033	7	0.0005			
Lack of fit	0.0024	3	0.0008	3.46	0.1306	not significant
Pure error	0.0009	4	0.0002			
Cor total	0.0542	16				

**Table 4:** Comparison of the model predictions with the experimental results.

Target Parameters	Projected Value	Actual Value	Relative Error
Surface roughness (nm)	0.803	0.78	2.86%

## Data Availability

The original contributions presented in this study are included in the article. Further inquiries can be directed to the corresponding author.
